# Psychometric analysis of ProQOL-BR in nursing: building hospital safety and protection

**DOI:** 10.1590/0034-7167-2024-0085

**Published:** 2024-12-16

**Authors:** Frederico Marques Andrade, Lanuza Borges Oliveira, Igor Monteiro Lima Martins, Maria Eduarda Borges Rodrigues, Carla Silvana de Oliveira e Silva

**Affiliations:** I Universidade Estadual de Montes Claros. Montes Claros, Minas Gerais, Brazil; II Centro Universitário FIPMoc. Montes Claros, Minas Gerais, Brazil

**Keywords:** Nursing Staff, Quality of Professional Life, Burnout, Validation Studies as Topic, Health Status Indicators., Profesionales de Enfermería, Calidad de Vida Profesional, Burnout, Estudios de Validación, Evaluación en Salud.

## Abstract

**Objectives:**

to analyze the psychometric properties of the ProQOL-BR instrument in hospital nursing professionals.

**Methods:**

a methodological study to validate the ProQOL-BR. Confirmatory factor analysis, assessment of local and global adjustment quality, Pearson hypothesis testing and Cronbach’s alpha internal consistency analysis were used.

**Results:**

a total of 490 professionals participated. The model presents adequate quality due to factor weights (λ≥ 0.40), acceptable overall fit quality and adequate chi-square ratio and degrees of freedom (χ2/g.1=2.51) for the parameters of CFI (0.923), GFI (0.902), TLI (0.914) and RMSEA (0.042). In terms of validity, it was shown to be adequate with CC=0.89. The internal consistency obtained by standardized Cronbach’s alpha was 0.761. Criterion validity was shown to be favorable with significant correlations (0.001).

**Conclusions:**

the instrument was validated regarding content, criteria and reliability. Three questions were removed from the original instrument, ProQOL-BR, leaving the final instrument with 25 questions.

## RESUMEN

**Objetivos:**

analizar las propiedades psicométricas del instrumento ProQOL-BR en profesionales de enfermería hospitalaria.

**Métodos:**

estudio de validación metodológica del ProQOL-BR. Se utilizaron análisis factorial confirmatorio, evaluación de la calidad del ajuste local y ajuste global, prueba de hipótesis de Pearson y análisis de consistencia interna alfa de Cronbach.

**Resultados:**

participaron 490 profesionales. El modelo presenta calidad adecuada debido a los pesos factoriales ((λ≥ 0,40), bondad de ajuste global aceptable y relación chi-cuadrado y grados de libertad (χ2/g.1=2,51) adecuados para los parámetros CFI (0,923), GFI (0,902), TLI (0,914) y RMSEA (0,042). En validez resultó adecuada con CC=0,89. La consistencia interna obtenida mediante el alfa de Cronbach estandarizado fue de 0,761. La validez de criterio fue favorable con correlaciones significativas (0,001).

**Conclusiones:**

el instrumento se encuentra validado en su contenido, criterio y confiabilidad. Del instrumento original, ProQOL-BR, se eliminaron tres preguntas, quedando el instrumento final con 25 preguntas.

## INTRODUCTION

The increased complexity of nursing service production has caused several emotional changes (EC) in its professionals around the world, thus creating the need to improve mechanisms that respond to these scenarios in modern society. Topics related to nursing professionals’ EC have been gaining prominence in discussions in various work environments, such as hospital settings^(1)^.

The improvement of these measures and concerns regarding the issue of EC are gaining initiatives in various scenarios, and one of these initiatives is the Sustainable Development Goals (SDGs): a list of 17 objectives and 169 targets to be achieved by 2030. They also have a call for health companies to act in the face of challenges to society’s sustainable development. In this context, it is important to highlight SDG 8 (Decent work and economic growth), with target 8.8, which aims to protect labor rights and promote safe and secure work environments for all workers^(2)^.

The emotional demands inherent to nursing in its work environment, such as living with human suffering, pain, death, direct and prolonged contact with patients, become a source of additional mental burden^(3,4)^. With nursing providing care as its main component of work, it must manage patients’ emotions, the suffering inherent in the health-disease processes as well as the emotional experience of caring itself^(5)^.

To understand nursing professionals’ EC, it is necessary to understand what professional quality of life (PQoL) is and how these changes are measured. Figley’s PQoL model^(6)^ underpins our entire understanding of EC. In this model, two poles are postulated: Compassion Satisfaction and Compassion Fatigue. Compassion Satisfaction refers to the rewards and personal achievements obtained through caring for others, whereas Compassion Fatigue is composed of two main elements: Burnout and Secondary Traumatic Stress^(7)^. Burnout manifests as a combination of emotional exhaustion, depersonalization, and reduced personal accomplishment, significantly affecting healthcare professionals’ quality of life and well-being. Secondary Traumatic Stress involves the emotional stress that professionals experience when exposed to their patients’ traumatic stories, whereas Compassion Fatigue encompasses a broader spectrum of negative experiences, referred to by Figley as “bad stuff”^(6)^. These experiences are subjective and vary according to individual perception, and are therefore not “natural” behaviors and emotions, but rather complex theoretical constructs^(8)^.

To measure these EC, we have the Professional Quality of Life Scale (ProQOL) instrument, originally developed by Stamm^(9)^ to measure the PQoL factors theoretically proposed by Figley. This instrument was adapted to the Brazilian context by Kennyston Lago and Vanderley Codo, resulting in ProQOL-BR^(10)^. This instrument assesses three main dimensions: Compassion Satisfaction, Burnout and Secondary Traumatic Stress, allowing an in-depth understanding of the emotional and psychological impacts experienced by healthcare professionals. The ProQOL-BR is a reduced and validated version for healthcare professionals in Brazil of the original instrument developed by Stamm^(9)^.

Statistical and psychometric analyses (especially validity and reliability) are necessary to develop assessment instruments in health. These analyses are essential to ensure the quality of results^(11)^. Failure to use valid and reliable instruments contributes to the reading of inaccurate or biased results, which may, consequently, compromise their application for the benefit of the population^(12)^.

Based on the subjective nature of the constructs and the diversity of guidelines on the subject, a group of experts developed a standardization study, recognized as the COSMIN initiative (COnsensus-based Standards for the selection of health Measurement INstruments), which is a definition of standards and concepts based on consensus of 57 experts from various parts of the world. The taxonomy proposed by COSMIN comprises three domains (validity, reliability and responsiveness), which also had their concepts internationally agreed upon^(13)^.

In Brazil, studies indicate that nursing workers are underprivileged^(14)^ and therefore more likely to have their quality of life impaired. EC and PQoL assessments can provide healthcare services with support to understand nursing professionals’ emotional burden, enabling the development of human resources policies to preserve the workforce and recover professionals affected by this syndrome.

## OBJECTIVES

To analyze the psychometric properties of the ProQOL-BR instrument from the perspective of nursing professionals working in highly complex hospital sectors.

## METHODS

### Ethical aspects

This research complies with the provisions of Resolution 466 of 2012. The research was approved by the *Universidade Estadual de Montes Claros* (UNIMONTES) Research Ethics Committee (REC). The Informed Consent Form was obtained from all individuals involved in the study by signing two copies: one for the researchers and one for participants.

### Study design, period and location

This is a methodological study to validate an instrument, the Professional Quality of Life Scale-Brazil (ProQOL-BR), to assess the phenomenon of Compassion Fatigue in nursing professionals working in a high-complexity hospital care unit. The study was conducted in four stages: systematic literature review; construct validity; criterion validity; and reliability analysis. The translated version of ProQOL-BR was used^(8)^.

The instrument was validated from July 2019 to March 2022, in the city of Montes Claros, state of Minas Gerais, Brazil.

### Population or sample; inclusion and exclusion criteria

The population consisted of 1,378 nursing professionals from highly complex hospital services in the northern macro-region of Minas Gerais. Sample size was calculated based on the total population, aiming to estimate population parameters with a 50% prevalence, a 95% confidence level, and a 5% accuracy. The participation of at least 301 nursing professionals was estimated. Sample selection was performed based on the simple random sampling technique. A list of all healthcare professionals in the northern macro-region of Minas Gerais was drawn up, numbered according to the number of elements, and, subsequently, a random draw was made by recomposing 600 professionals to be invited.

Professionals with more than six months of work in the sector and who were active at the time of data collection were included. Professionals who were away from work due to leave, for any reason, or on vacation were excluded.

### Study protocol

Several factors contributed to the choice of ProQOL-BR^(9)^ as the instrument to be applied. One of the factors is that it is an instrument that assesses PQoL through the analysis of Compassion Fatigue, associating high Burnout and Secondary Traumatic Stress with decreased Compassion Satisfaction. This is one of the multidimensional instruments for assessing emotional state most used in research, but ProQOL-BR is validated for Brazilian healthcare professionals, not for foreign nursing professionals.

Before starting data collection, each institution was asked for authorization. Then, professionals were made aware of the need to participate in the research. After each professional signed a consent form, a questionnaire was given to be completed at home, informing them of the return date. The questionnaires were collected on professionals’ next work day.

In addition to ProQOL-BR, other instruments were applied to comply with the psychometric validity protocol. The Work Stress Scale (WSS) was applied to assess stress at work. The Beck Anxiety Inventory (BAI), adapted and validated for Portuguese, was used to assess the characteristic symptoms of anxiety. Another questionnaire used was the Dispositional Resilience (Hardiness) Scale, originally from the United States of America, which aims to assess the degree to which people have “hardy” attitudes when facing stressful situations. Initially, the questionnaire consisted of a sociodemographic questionnaire to verify associations between the instruments and the population characteristics.

The ProQOL-BR is a scale composed of 30 items, distributed in three dimensions: Compassion Satisfaction, Compassion Fatigue and Burnout. These dimensions provide a comprehensive view of healthcare professionals’ well-being and mental health. The WSS is composed of 23 items and measures occupational stress through three dimensions: Pressure at Work, Control over Activities and Social Support. This instrument allows the identification of stressful conditions that affect workers’ productivity and health. The BAI contains 21 items that assess the severity of anxiety, addressing common physical and emotional symptoms, and is widely used for diagnosis and therapeutic monitoring. Finally, the Dispositional Resilience (Hardiness) Scale, composed of 45 items, measures psychological resilience through three components, such as Control, Commitment and Challenge, assessing individuals’ ability to adapt to stressful situations. These instruments are fundamental in mental health research and clinical practice, providing essential data for developing intervention strategies and health promotion in the workplace.

The use of these instruments was essential to establish their convergent-discriminant validity. These instruments, recognized and widely used in the literature, share similarities in the assessment of psychological and emotional aspects related to well-being, stress and resilience, allowing us to verify whether the new instrument measures similar constructs (convergent validity) or distinct ones (discriminant validity). For instance, the positive correlation between the new instrument and ProQOL-BR may indicate that it effectively measures PQoL, whereas a negative correlation with the WSS may demonstrate its ability to discriminate between well-being and occupational stress. Likewise, comparisons with the BAI and the Dispositional Resilience (Hardiness) Scale may reinforce the validity of the new instrument by showing consistent relationships with measures of anxiety and resilience. These instruments establish a robust criterion for assessing the new instrument’s accuracy and specificity, ensuring that it is a reliable and effective tool in measuring the psychological constructs in question.

The ProQOL-BR was assessed for validity and reliability, following three stages: 1) Construct validity through exploratory factor analysis (EFA), ratified by confirmatory factor analysis (CFA) and convergent validity, estimated by composite reliability (CR). Construct validity aims to verify whether the instrument’s structure can measure the theoretical construct it intends to; 2) Concurrent criterion validity with Pearson hypothesis testing. Criterion assessment seeks to validate whether the instrument quantifies the strength and nature of the relationship between the instrument’s measures and external criteria, providing evidence on the instrument’s validity in different contexts and periods of application; 3) Reliability analysis, through Cronbach’s alpha internal consistency analysis. Reliability analysis aims to verify whether results are consistent and stable, increasing confidence in the interpretation of the scores obtained by participants.

### Analysis of results, and statistics

For construct validity, CFA was used to ratify the dimensional structure extracted by EFA. As an indicator of local adjustment quality, the factorial weight (λ≥0.40)^(15)^ was used and, to assess the adjusted measurement model’s overall adjustment quality, chi-square ratio and degrees of freedom (χ2/g.1), goodness-of-fit index (GFI), Tucker-Lewis index (TLI), Bentler comparative adjustment index (CFI) and root mean square error of approximation (RMSEA) were used. The parameters considered to assess the global adjustment of the model were λ2/g.1<5, CFI, GFI, TLI≥0.9 and RMSEA <0.10^(15)^, and to assess convergent validity, CC≥0.70 was used^(16)^.

The purpose of concurrent criterion validity was to determine whether ProQOL-BR was capable of detecting a higher incidence of Compassion Fatigue in nursing professionals, who were classified with a higher BAI score, a higher WSS score and with incidences on the Dispositional Resilience (Hardiness) Scale. This validity was performed using Pearson hypothesis test.

Reliability analysis was conducted by analyzing internal consistency using Cronbach’s alpha coefficient and item/total correlation using the correlation matrix of all items in the instrument. The ideal alpha value range was considered to be between 0.7 and 0.9^(17)^.

## RESULTS

A total of 490 nursing professionals participated in this study. ProQOL-BR descriptive analysis was performed using the distribution of absolute and relative frequencies, in addition to the mean classification of the three dimensions based on the instrument’s 28 items (Table 1). The professionals presented a mean of Compassion Fatigue.

**Table 1:** Average scores of the Professional Quality of Life Scale-Brazil factors of nursing professionals working in more complex hospital services in northern Minas Gerais, Montes Claros, Minas Gerais, Brazil, 2024 (N=490)

Factors	Items	M
Compassion Satisfaction	Q1 - I am happy	3.99
	Q3 - I get satisfaction from being able to [help] people	4.42
	Q6 - I feel invigorated after working with those I [help]	3.56
	Q12 - I like my work as a [helper]	4.39
	Q16 - I am pleased with how I am able to keep up with [helping] techniques and protocols	4.14
	Q17 - I am the person I always wanted to be	3.81
	Q18 - My work makes me feel satisfied	3.96
	Q20 - I have happy thoughts and feelings about those I [help] and how I could help them	4.00
	Q22 - I believe I can make a difference through my work	4.22
	Q24 - I am proud of what I can do to [help]	4.36
	Q27 - I have thoughts that I am a “success” as a [helper]	3.42
	Q30 - I am happy that I chose to do this work	4.36
Secondary Traumatic Stress	Q5 - I jump or am startled by unexpected sounds	2.55
	Q7 - I find it difficult to separate my personal life from my life as a [helper]	2.28
	Q8 - I am not as productive at work because I am losing sleep over traumatic experiences of a person I [help]	1.50
	Q9 - I think that I might have been affected by the traumatic stress of those I [help]	1.81
	Q10 - I feel trapped by my job as a [helper]	1.63
	Q11 - Because of my [helping], I have felt “on edge” about various things	2.25
	Q13 - I feel depressed because of the traumatic experiences of the people I [help]	2.03
	Q14 - I feel as though I am experiencing the trauma of someone I have [helped]	1.81
	Q23 - I avoid certain activities or situations because they remind me of frightening experiences of the people I [help]	1.72
	Q25 - As a result of my [helping], I have intrusive, frightening thoughts	1.48
Burnout	Q19 - I feel worn out because of my work as a [helper]	2.54
	Q21 - I feel overwhelmed because my work load seems endless	2.48
	Q26 - I feel “bogged down” by the system	2.53

The Compassion Satisfaction dimension obtained an overall average score of 59.48 points, classifying it as “average Compassion Satisfaction”. In the group with lower factors, “Q27 - I have thoughts that I am a “success” as a [helper]” stood out (M= 3.42). The questions that presented the highest factors were “Q3 - I get satisfaction from being able to [help] people” (M= 4.42), followed by “Q12 - I like my work as a [helper]” (M= 4.39), “Q24 - I am proud of what I can do to [help]” and “Q30 - I am happy that I chose to do this work” (both with M= 4.36).

The Secondary Traumatic Stress dimension obtained an overall average score of 19.06 points, classifying it as “low Secondary Traumatic Stress”. The questions that presented the highest factors were “Q5 - I jump or am startled by unexpected sounds” (M= 2.55), “Q7 - I find it difficult to separate my personal life from my life as a [helper]” (M= 2.28) and “Q11 - Because of my [helping], I have felt “on edge” about various things” (M= 2.25). In the group with lower factors, “Q25 - As a result of my [helping], I have intrusive, frightening thoughts” (M= 1.48) and “Q8 - I am not as productive at work because I am losing sleep over traumatic experiences of a person I [help]” (M= 1.50) stand out.

The Burnout dimension obtained an overall average score of 7.55 points, classifying it as “medium Burnout”. The question with the highest factor was “Q19 - I feel worn out because of my work as a [helper]” (M= 2.54). The question with the lowest factor was “Q21 - I feel overwhelmed because my work load seems endless” (M= 2.48). It is worth noting that the variation in this dimension was very small (0.06 points), thus representing a balance between the factors for Burnout.

**Figure 1:**
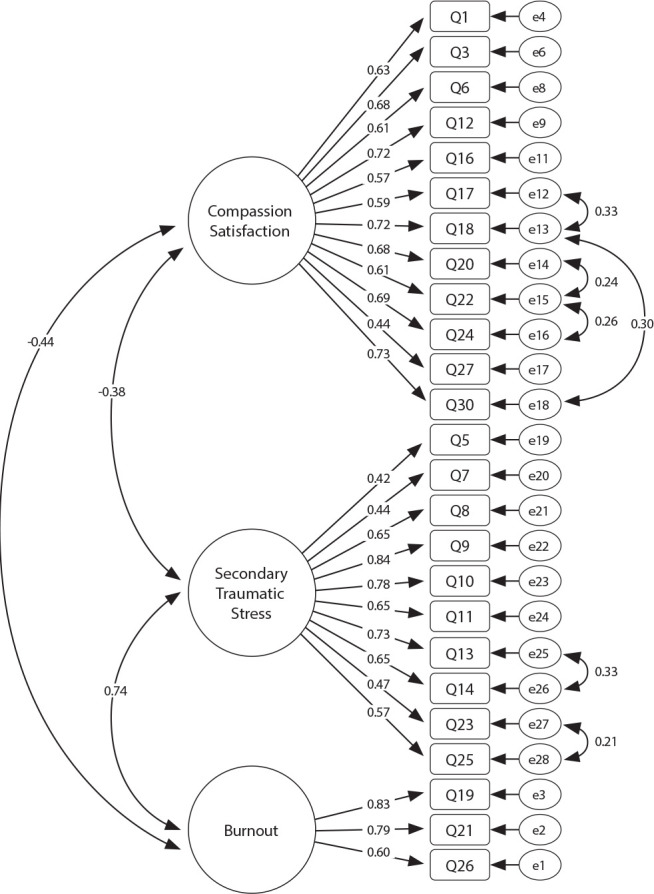
Professional Quality of Life Scale-Brazil model ratified by confirmatory factor analysis, Montes Claros, Minas Gerais, Brazil, 2024 (N=490)

The proposed model ratified by CFA presented adequate quality of local adjustment by the factorial weights (λ≥ 0.40) in 25 questions (Figure 1). Furthermore, three questions - Q2 (λ=0.09), Q4 (λ=0.35) and Q15 (λ=0.12) - from ProQOL-BR were removed, as they presented low factor loadings for nursing professionals. The model also presented acceptable overall goodness-of-fit by the chi-square ratio and degrees of freedom (χ2/g.1=2.51) and suitable for the parameters of CFI (0.923), GFI (0.902), TLI (0.914) and RMSEA (0.042). In convergent validity assessment, the proposed model also proved to be suitable, with CC=0.89.

The reliability results of ProQOL-BR reveal that the internal consistency obtained from standardized Cronbach’s alpha was 0.761 for the entire sample and from 0.768 to 0.860 for the instrument dimensions. Cronbach’s alpha values, if a given item were excluded, were adequate in 26 items, since all items presented values from 0.801 to 0.868. Two Burnout dimension items presented indexes close to adequate if they were excluded (Q-19 0.631 and Q-21 0.629), but were maintained due to the index and the number of dimension items. The corrected item-total correlations performed using Pearson’s correlation coefficient were weak for item “Q2 - I am preoccupied with more than one person I [help]” (r=0.127) and strong for item “Q10 - I feel trapped by my job as a [helper]” (r=0.667) (Table 2).

**Table 2:** Analysis of the internal consistency of the Professional Quality of Life Scale-Brazil using Cronbach’s alpha coefficient and corrected item-total correlation, Montes Claros, Minas Gerais, Brazil, 2024 (N=490)

Dimensions of ProQOL-BR	ProQOL-BR Item	Cronbach’s alpha (95% CI)	Cronbach’s alpha if item is removed	Item-Total Corelation
Compassion Satisfaction	Q1 - I am happy	0.845	0.831	0.571
	Q3 - I get satisfaction from being able to [help] people		0.830	0.638
	Q6 - I feel invigorated after working with those I [help]		0.831	0.546
	Q12 - I like my work as a [helper]		0.827	0.661
	Q16 - I am pleased with how I am able to keep up with [helping] techniques and protocols		0.833	0.528
	Q17 - I am the person I always wanted to be		0.831	0.539
	Q18 - My work makes me feel satisfied		0.824	0.664
	Q20 - I have happy thoughts and feelings about those I [help] and how I could help them		0.826	0.660
	Q22 - I believe I can make a difference through my work		0.830	0.592
	Q24 - I am proud of what I can do to [help]		0.830	0.611
	Q27 - I have thoughts that I am a “success” as a [helper]		0.840	0.410
	Q30 - I am happy that I chose to do this work		0.826	0.638
Secondary Traumatic Stress	Q5 - I jump or am startled by unexpected sounds	0.860	0.860	0.427
	Q7 - I find it difficult to separate my personal life from my life as a [helper]		0.863	0.429
	Q8 - I am not as productive at work because I am losing sleep over traumatic experiences of a person I [help]		0.847	0.582
	Q9 - I think that I might have been affected by the traumatic stress of those I [help]		0.830	0.758
	Q10 - I feel trapped by my job as a [helper]		0.838	0.667
	Q11 - Because of my [helping], I have felt “on edge” about various things		0.847	0.565
	Q13 - I feel depressed because of the traumatic experiences of the people I [help]		0.834	0.709
	Q14 - I feel as though I am experiencing the trauma of someone I have [helped]		0.839	0.653
	Q23 - I avoid certain activities or situations because they remind me of frightening experiences of the people I [help]		0.855	0.463
	Q25 - As a result of my [helping], I have intrusive, frightening thoughts		0.849	0.542
Burnout	Q19 - I feel worn out because of my work as a [helper]	0.768	0.631	0.657
	Q21 - I feel overwhelmed because my work load seems endless		0.629	0.654
	Q26 - I feel “bogged down” by the system		0.801	0.506
Total score		0.761		

The concurrent criterion validity of ProQOL-BR was favorable for nursing professionals (Table 3). The significance of the correlations was significant (0.001), with some medium magnitudes and most strong. All ProQOL-BR dimensions with the Dispositional Resilience (Hardiness) Scale (Commitment and Control) dimensions showed a strong magnitude of correlation, ranging from 0.335 to 0.484. The correlation of ProQOL-BR dimensions with the Dispositional Resilience (Hardiness) Scale (Challenge) dimension was of medium magnitude, ranging from 0.202 to 0.268. The correlation of ProQOL-BR dimensions with WSS showed a strong magnitude of correlation, ranging from 0.350 to 0.597. The correlation of ProQOL-BR dimensions with BAI showed a strong magnitude of correlation in the Secondary Traumatic Stress and Burnout dimensions, ranging from 0.426 to 0.493, with an average magnitude of correlation in the Compassion Satisfaction dimension, with a value of 0.218.

**Table 3:** Pearson correlations between the measures obtained by Professional Quality of Life Scale-Brazil, Dispositional Resilience (Hardiness) Scale, Work Stress Scale and Beck Anxiety Inventory

Scales	ProQOL-BR		
	D1 (Satisfaction) *r* (*p* value)	D2 (Estresse) *r* (*p* value)	D3 (Burnout) *r* (*p* value)
Dispositional Resilience (Hardiness) Scale (Commitment)	0.484 (<0.001)	-0.462 (<0.001)	-0.469 (<0.001)
Dispositional Resilience (Hardiness) Scale (Control)	0.405 (<0.001)	-0.394 (<0.001)	-0.335 (<0.001)
Dispositional Resilience (Hardiness) Scale (Challenge)	0.210 (<0.001)	-0.202 (<0.001)	-0.268 (<0.001)
Total Work Stress Scale	-0.350 (<0.001)	0.470 (<0.001)	0.597 (<0.001)
Beck Anxiety Inventory	-0.218 (<0.001)	0.493 (<0.001)	0.426 (<0.001)

--------------

## DISCUSSIONS

This study provided results that demonstrated ProQOL-BR construct validity, reliability and criteria in the population of nursing professionals working in highly complex hospital care units. These results were consistent and reliable. The instrument is important for directing the detection of EC in this population, in addition to serving to achieve SDG 8 (Decent work and economic growth), aiming at protecting labor rights and promoting safer work environments for nursing professionals^(2)^. National literature indicates that nursing is the leading actor of any health company. Its deliveries impact costs, customer satisfaction and, mainly, the ultimate goal of this company and the reestablishment of the health conditions of those who seek the service^(18)^.

This study showed a high participation rate in the survey. Moreover, 82% of invited professionals answered the questionnaires, demonstrating that the topic is of interest and relevance to this category. It is noteworthy that, especially after the COVID-19 pandemic period, nursing professionals have been very concerned about their emotional situation^(19)^. Concern about EC issues becomes essential for the creation of an effective and humane health system.

The ProQOL-Br enables several important analyses of nursing professionals’ quality of life at work, resulting in a global analysis of Compassion Fatigue. In the Compassion Satisfaction dimension, the results indicated “average Compassion Satisfaction”. There was a balance resulting from low rates in relation to the success of the consequences of their care and high rates in relation to the satisfaction of being a nursing professional. This result may be related to the severity of the patients to whom these professionals provide care, an environment full of deaths and palliative care, but also that provides “pride” in being able to use their knowledge and skills to do the best for human beings in such critical health situations^(20)^.

Regarding Secondary Traumatic Stress, which refers to invasive and frightening thoughts resulting from the care provided, the instrument indicated “low secondary stress”, one of the questions that gave the lowest results in the nursing professionals in the study. Secondary Traumatic Stress was developed to measure symptoms specifically in professionals who provide care. Similar results to this study of Secondary Traumatic Stress were indicated in a validity study that showed results with firefighters and paramedics^(21)^.

The Burnout dimension presented “medium burnout” results. A study^(22)^ used the Maslach Burnout Inventory (MBI), validated in Brazil, to assess this condition in nursing professionals working in intensive care. This study also indicated a low average for burnout in its population. The same study recalls that burnout rates in Brazilian nursing professionals are lower compared to European ones.

The nursing professionals in this study did not present Compassion Fatigue. The results are similar to those found in a study in the state of Paraná^(23)^. These data may be related to resilience at work. In the results and discussions of this study in Paraná, resilience was shown to be a protective factor for mental health variables, such as the presence of minor psychological disorders, emotional exhaustion and depersonalization^(23)^.

CFA indicated an adequate favorable instrument adjustment in relation to the population of nursing professionals working in intensive care. The factorial loads with better adjustments indicated that professionals feel happy to have chosen nursing as a profession, but at the same time recognize how much they are affected by the stress of the care process, which causes exhaustion. The results are the same as those of a study of the EC of nursing professionals who worked in intensive care with patients with COVID-19^(24)^. These professionals feel emotionally exhausted, as do their teammates. The origin of emotional exhaustion is related to clinical instability, high number of deaths and frequency of institutional changes.

The results showed the instrument reliability for all its dimensions. The most notable values indicated that nursing professionals working in intensive care have difficulty separating their personal and professional lives, and that, in environments outside of work, they avoid certain activities or situations that may recall unpleasant experiences they have had with their patients.

The analyses indicated concurrent criterion validity, with the ProQOL-BR being able to verify a higher incidence of Compassion Fatigue in nursing professionals who were classified with a higher value for anxiety (BAI) and a higher value for work stress (WSS). Anxiety and stress were highlighted in this study, as indicated in the literature. In a Brazilian study^(25)^, the vast majority (75%) of professionals presented mental distress, highlighting symptoms of depression, anxiety and stress in the execution of their work routines.

The criterion validity was also favorable with the instrument on hardiness personality. The data obtained with this instrument showed that the greater the coping behavior in adverse situations, the better the indices related to Compassion Satisfaction and the worse the indices for Stress and Burnout. The professionals in this study presented satisfactory indices for hardiness personality in its three dimensions, which was also found in a study^(26)^ carried out with professionals from a highly complex company, demonstrating that hardiness personality directly and indirectly influences health and well-being, promoting the use of social resources and altering self-perceived stress, thus reducing tension in health work.

### Study limitations

Despite the relevance of the instrument developed, its limitations must be considered. Few national studies have applied the methodology proposed in ProQOL-Br^(8)^ validity to interpret its results. Based on these results, the need to update value analysis by nursing professionals is highlighted.

### Contributions to nursing, health or public policy

It is expected that the use of this instrument will enable the implementation of actions aimed at harmful EC intervention processes in nursing professionals as well as the identification of the processes’ potentialities and weaknesses. Furthermore, it is expected that the application of this tool will assist managers and healthcare professionals in structuring their human resources policies and in all interpersonal relationships existing in an environment as complex and full of distinct nuances as nursing care for critically ill patients.

## CONCLUSIONS

The methodological development for ProQOL-Br analysis and validity for nursing professionals working in highly complex healthcare companies made it possible to understand a topic that is difficult to measure: emotional issues. This understanding and validity contribute significantly to any intervention on the topic.

The instrument has been validated in terms of its content, criteria and reliability. Three questions were removed from the original instrument, ProQOL-BR, leaving the final instrument with 25 questions. It is important to note that new analyses should be assessed. The instrument is available for use in studies, and further analyses may be useful in improving it.

For EC assessment in Brazilian nursing professionals, the use of this instrument is recommended, since its validity occurred with professionals during a pandemic care period, and the number of questions makes its application quick and objective.

## References

[1] Pereira MD, Torres EC, Pereira MD, Antunes PFS, Costa CFT. (2020). Sofrimento emocional dos enfermeiros no context hospitalar frente à pandemia COVID-19. RSD.

[2] Ferreira TC, Caldana ACF, Batalhão AC, Alves MFR, Paliari JC. (2023). Objetivos de Desenvolvimento Sustentável: o impacto de grandes representantes da construção brasileira. Amb Soc.

[3] Souza HA, Bernardo MH. (2019). Prevenção de adoecimento mental relacionado ao trabalho: a práxis de profissionais do Sistema Único de Saúde comprometidos com a saúde do trabalhador. Rev Bras Saúde Ocup.

[4] Pinhatti EDG, Ribeiro RP, Soares MH, Martins JT, Lacerda MR. (2018). Distúrbios psíquicos menores na enfermagem: prevalência e fatores associados. Rev Bras Enferm.

[5] Yasin JCM, Barlem ELD, Ruivo ÉDG, Andrade GB, Silveira RS, Bremer LCF. (2023). Problemas éticos vividos por enfermeiros durante a COVID-19: relações com o sofrimento mental. Texto Contexto Enferm.

[6] Figley CR. (1995). Compassion fatigue: coping with secondary traumatic stress disorder in those who treat the traumatized.

[7] Figley CR. (2002). Treating compassion fatigue.

[8] Jenkins SR, Baird S. (2002). Secondary traumatic stress and vicarious trauma: a validational study. J Trauma Stress.

[9] Stamm BH. (2005). The ProQOL Manual.

[10] Lago K, Codo W. (2013). Fadiga por compaixão: evidências de validade fatorial e consistência interna do ProQol-BR. Estud Psicol (Natal).

[11] Streiner D, Norman G, Cairney J. (2015). Health measurement scales: a practical guide to their development and use.

[12] Souza AC, Alexandre NMC, Guirardello EB. (2017). Propriedades psicométricas na avaliação de instrumentos: avaliação da confiabilidade e da validade. Epidemiol Serv Saúde.

[13] Mokkink LB, Prinsen CAC, Patrick DL, Alonso J, Bouter LM, Vet HC (2019). COSMIN: Study Design checklist for Patient-reported outcome measurement instruments.

[14] Silva MCN, Machado MH. (2020). Sistema de Saúde e Trabalho: desafios para a Enfermagem no Brasil. Ciênc Saúde Coletiva.

[15] Valentini F, Damásio BF. (2016). Variância Média Extraída e Confiabilidade Composta: Indicadores de Precisão. Psicol: Teor Pesq.

[16] Espinoza V, Maritza SA, Olivia RE, Noé SCK. (2015). Validação do construto e da confiabilidade de uma escala de inteligência emocional aplicada a estudantes de enfermagem. Rev Latino-Am Enfermagem.

[17] Marôco JP, Campos JADB, Vinagre MG, Pais-Ribeiro JL. (2014). Adaptação Transcultural Brasil-Portugal da Escala de Satisfação com o Suporte Social para Estudantes do Ensino Superior. Psicol Reflex Crit.

[18] Galon T, Navarro VL, Gonçalves AMS. (2022). Percepções de profissionais de enfermagem sobre suas condições de trabalho e saúde no contexto da pandemia de COVID-19. Rev Bras Saúde Ocup.

[19] Lima ACO, Santos BOS, Nascimento BTS, Mesquita CM, Paz NC, Moreno TR (2023). O impacto da pandemia de COVID-19 na saúde mental dos profissionais de enfermagem no âmbito hospitalar: uma revisão integrativa da literatura. Braz J Health Rev.

[20] Silva CMV, Silva JCP, Santos DAC, Vieira DA, Marques SLL, Nojosa SS (2023). Cuidados de enfermagem a pacientes com COVID-19 em Unidade de Terapia Intensiva: uma revisão integrativa. RSD.

[21] Dalagasperina P, Castro EK, Cruz RM, Pereira A, Jiménez BM. (2021). Estrutura Interna da Versão Brasileira do Questionário de Estresse Traumático Secundário. Psicol USF.

[22] Vieira LS, Machado WL, Dal Pai D, Magnago TSBS, Azzolin KO, Tavares JP. (2022). Burnout e resiliência em profissionais de enfermagem de terapia intensiva frente à COVID-19: estudo multicêntrico. Rev Latino-Am Enfermagem.

[23] Fabri NV, Martins JT, Galdino MJQ, Ribeiro RP, Moreira AAO, Haddad MCLF. (2021). Satisfação, fadiga por compaixão e fatores associados em enfermeiros da atenção básica. Enferm Glob.

[24] Ampos LF, Vecchia LPD, Tavares JP, Camatta MW, Magnago TSBS, Pai DD. (2023). Implicações da atuação da enfermagem no enfrentamento da COVID-19: exaustão emocional e estratégias utilizadas. Esc Anna Nery.

[25] Secchi Á, Scortegagna SA, Kantorski LP. (2023). Grupos balint com o uso do aplicativo m-health coletivos em saúde mental na COVID-19. Texto Contexto Enferm.

[26] Silva-Junior RF, Alves ECS, Santos KO, Santos SP, Barbosa HA, Siqueira LG (2020). Personalidade hardiness e fatores associados em profissionais da saúde atuantes em serviços que atendem pacientes críticos. Ciênc Saúde Coletiva.

