# Supplementary material for: Psychometric analysis of ProQOL-BR in nursing: building hospital safety and protection
Source: Rev Bras Enferm. 2024 Dec 16;77(6):e20240085. doi: 10.1590/0034-7167-2024-0085 (PMC11654526; doi:10.1590/0034-7167-2024-0085)
Supplement: Translation

# Translation: Análise psicométrica do instrumento ProQOL-BR em enfermagem: construindo segurança e proteção hospitalar

**DOI:** 10.1590/0034-7167-2024-0085pt

**Authors:** Frederico Marques Andrade, Lanuza Borges Oliveira, Igor Monteiro Lima Martins, Maria Eduarda Borges Rodrigues, Carla Silvana de Oliveira e Silva

**Affiliations:** I Universidade Estadual de Montes Claros. Montes Claros, Minas Gerais, Brasil; II Centro Universitário FIPMoc. Montes Claros, Minas Gerais, Brasil

**Keywords:** Profissionais de Enfermagem, Qualidade de Vida Profissional, Esgotamento Emocional, Estudos de Validação, Avaliação em Saúde.

## Abstract

**Objetivos:**

analisar as propriedades psicométricas do instrumento ProQOL-BR em profissionais de enfermagem hospitalares.

**Métodos:**

estudo metodológico de validação do ProQOL-BR. Utilizaram-se análise fatorial confirmatória, avaliação da qualidade de ajuste local e ajuste global, teste de hipóteses Pearson e análise de consistência interna alfa de Cronbach.

**Resultados:**

participaram 490 profissionais. O modelo apresenta qualidade adequada pelos pesos fatoriais (λ≥ 0,40), qualidade de ajuste global aceitável e razão qui-quadrado e graus de liberdade (2/g.1=2,51) adequada para os parâmetros de CFI (0,923), GFI (0,902), TLI (0,914) e RMSEA (0,042). Na validade, mostrou-se adequado com CC=0,89. A consistência interna obtida pelo alfa de Cronbach padronizado foi de 0,761. A validade de critério mostrou-se favorável com correlações significativas (0,001).

**Conclusões:**

o instrumento encontra-se validado em seu conteúdo, critério e confiabilidade. Do instrumento original, ProQOL-BR, foram retiradas três questões, ficando o instrumento final com 25 questões.

## INTRODUÇÃO

O aumento da complexidade de produção do serviço de enfermagem tem causado diversas alterações emocionais (AE) em seus profissionais pelo mundo, nascendo, assim, a necessidade de aprimorar mecanismos que respondam a esses cenários da sociedade moderna. Temáticas relacionadas às AE dos profissionais de enfermagem vêm tomando destaque nas discussões em diversos ambientes de trabalho, como o ambiente hospitalar^(1)^.

O aprimoramento dessas medidas e preocupações quanto à temática sobre AE ganha iniciativas em diversos cenários, e uma dessas iniciativas são os Objetivos de Desenvolvimento Sustentável (ODS): uma lista com 17 objetivos e 169 metas a serem atingidas até 2030. Possuem, também nas empresas de saúde, um chamamento a agir perante desafios para o desenvolvimento sustentável da sociedade. Nesse contexto, é importante destacar o ODS 8 (Trabalho descente e crescimento econômico), com a meta 8.8, que visa proteger os direitos trabalhistas e promover ambientes de trabalho seguros e protegidos para todos os trabalhadores^(2)^.

As exigências emocionais inerentes à enfermagem no seu ambiente de trabalho, como o convívio com o sofrimento humano, a dor, a morte, o contato direto e prolongado com os pacientes, tornam-se uma fonte de carga mental adicional^(3,4)^. Tendo a enfermagem o cuidar como seu principal componente de trabalho, ela deve gerir as emoções do cliente, o sofrimento inerente aos processos de saúde-doença, bem como a própria experiência emocional de cuidar^(5)^.

Para o entendimento das AE dos profissionais de enfermagem, faz-se necessário entender o que é a qualidade de vida profissional (QVP) e como são mensuradas essas alterações. O modelo de QVP de Figley^(6)^ fundamenta todo nosso entendimento acerca das AE. Nesse modelo, postulam dois polos: a Satisfação por Compaixão e a Fadiga por Compaixão. A Satisfação por Compaixão refere-se às recompensas e realizações pessoais obtidas através do cuidado aos outros, enquanto que a Fadiga por Compaixão é composta por dois elementos principais: *Burnout* e Estresse Traumático Secundário^(7)^. O *Burnout* se manifesta como uma combinação de exaustão emocional, despersonalização e redução da realização pessoal, afetando significativamente a qualidade de vida e o bem-estar dos profissionais de saúde. O Estresse Traumático Secundário envolve o estresse emocional que profissionais experienciam quando expostos às histórias traumáticas dos seus clientes, enquanto que a Fadiga por Compaixão engloba um espectro mais amplo de vivências negativas, referidas por Figley como “coisas ruins” (*bad stuff*)^(6)^. Essas experiências são subjetivas e variam conforme a percepção individual, não sendo, portanto, comportamentos e emoções “naturais”, mas sim construtos teóricos complexos^(8)^.

Para mensurar essas AE, temos o instrumento *Professional Quality of Life Scale* (ProQOL), originalmente desenvolvido por Stamm^(9)^ para medir os fatores de QVP teoricamente propostos por Figley. Esse instrumento foi adaptado para o contexto brasileiro por Kennyston Lago e Vanderley Codo, resultando no ProQOL-BR^(10)^. Esse instrumento avalia três dimensões principais: Satisfação por Compaixão, *Burnout* e Estresse Traumático Secundário, permitindo uma compreensão aprofundada dos impactos emocionais e psicológicos vivenciados por profissionais de saúde. O ProQOL-BR é uma versão reduzida e validada para profissionais de saúde no Brasil do instrumento original elaborado por Stamm^(9)^.

Para a construção de instrumentos de avaliação na área da saúde, são necessárias análises estatísticas e psicométricas (especialmente validade e confiabilidade). Essas análises são essenciais para a garantia da qualidade dos resultados^(11)^. A não utilização de instrumentos válidos e confiáveis contribui para a leitura de resultados imprecisos ou tendenciosos, que podem, por conseguinte, comprometer a aplicação deles em benefício da população^(12)^.

Tendo como base a natureza subjetiva dos construtos e a diversidade de orientações em relação ao tema, um grupo de especialistas desenvolveu um estudo de padronização, reconhecido como iniciativa COSMIN (*COnsensus-based Standards for the selection of health Measurement INstruments*), que se trata de uma definição de normas e conceitos baseados em consenso de 57 especialistas de várias partes do mundo. A taxonomia proposta pela COSMIN compreende três domínios (validade, confiabilidade e responsividade), que também tiveram seus conceitos acordados internacionalmente^(13)^.

No Brasil, trabalhos apontam condições não privilegiadas^(14)^ para os trabalhadores de enfermagem e, portanto, mais propensos a ter prejuízo na sua qualidade de vida. As avaliações das AE e QVP podem permitir aos serviços de saúde subsídios para o entendimento diante da carga emocional dos profissionais de enfermagem, possibilitando a construção de políticas de recursos humanos para a preservação da força de trabalho e recuperação de profissionais acometidos por esta síndrome.

## OBJETIVOS

Analisar as propriedades psicométricas do instrumento ProQOL-BR segundo a perspectiva de profissionais de enfermagem atuantes em setores hospitalares de alta complexidade.

## MÉTODOS

### Aspectos éticos

A presente pesquisa atende o que determina a Resolução nº 466 de 2012. A pesquisa obteve aprovação pelo Comitê de Ética em Pesquisa (CEP) da Universidade Estadual de Montes Claros (UNIMONTES). O Termo de Consentimento Livre e Esclarecido foi obtido de todos os indivíduos envolvidos no estudo por meio assinatura em duas vias: uma para os pesquisadores e outra para o participante.

### Desenho, período e local do estudo

Estudo metodológico de validação de um instrumento, o *Professional Quality of Life Scale-Brazil* (ProQOL-BR), para avaliação do fenômeno Fadiga por Compaixão em profissionais de enfermagem atuantes em unidade de assistência hospitalar de alta complexidade. O estudo foi realizado em quatro etapas: revisão sistemática da literatura; validade de construto; validade de critério; e análise da confiabilidade. Utilizou-se a versão traduzida do ProQOL-BR^(8)^.

A validação do instrumento ocorreu no período de julho de 2019 a março de 2022, na cidade de Montes Claros, estado de Minas Gerais, Brasil.

### População ou amostra; critérios de inclusão e exclusão

A população foi formada por 1.378 profissionais de enfermagem dos serviços hospitalares de alta complexidade da macrorregião Norte de Minas Gerais. O tamanho da amostra foi calculado com base na população total, visando estimar parâmetros populacionais com prevalência de 50%, nível de confiança de 95% e precisão de 5%. Estimou-se a participação de, no mínimo, 301 profissionais de enfermagem. A seleção da amostra foi realizada com base na técnica de amostragem aleatória simples. Foi elaborada uma lista de todos os profissionais de saúde da macrorregião Norte de Minas Gerais, numerados de acordo com a quantidade de elementos, e, posteriormente, feito o sorteio aleatório por recomposição de 600 profissionais a serem convidados.

Incluíram-se profissionais com mais de seis meses de trabalho no setor e estar atuante (ativo) no momento da coleta dos dados. Excluíram-se profissionais em afastamento das atividades laborais por licença, por qualquer motivo, em período de férias.

### Protocolo do estudo

Vários fatores contribuíram para a escolha do ProQOL-BR^(9)^ como instrumento a ser aplicado. Um dos fatores é por ser um instrumento que avalia QVP por meio de análise da Fadiga por Compaixão, associando *Burnout* e Estresse Traumático Secundário elevados com a diminuição da Satisfação por Compaixão. Esse é um dos instrumentos multidimensionais, para avaliação de estado emocional, mais utilizados em pesquisas, porém o ProQOL-BR é validado para profissionais de saúde brasileiros, não para profissionais de enfermagem estrangeiros.

Antes de iniciar a coleta de dados, foi solicitada a autorização de cada instituição. Em sequência, foram sensibilizados os profissionais para participação da pesquisa. Após o consentimento de cada profissional, por meio de assinatura de termo, foi entregue um questionário para ser respondido em domicílio, informando sobre a data de retorno. No próximo dia de trabalho do profissional, os questionários eram recolhidos.

Para atendimento ao protocolo de validação psicométrica, além do ProQOL-BR, foram aplicados outros instrumentos. Para aferição do estresse no trabalho, foi aplicada a Escala de Estresse no Trabalho (EET). Para avaliação dos sintomas característicos de ansiedade, foi utilizado o Inventário de Ansiedade de Beck (BAI), adaptado e validado para o português. Outro questionário utilizado foi a Escala de *Hardiness*, original dos Estados Unidos da América, que tem por finalidade avaliar o quanto de atitudes “*hardy*” as pessoas têm no enfrentamento de situações estressantes. Inicialmente, o questionário era constituído de um questionário sociodemográfico para verificação de associações entre os instrumentos e as características da população.

O ProQOL-BR é uma escala composta por 30 itens, distribuídos em três dimensões: Satisfação por Compaixão, Fadiga por Compaixão e *Burnout*. Essas dimensões fornecem uma visão abrangente do bem-estar e da saúde mental dos profissionais de saúde. A EET é composta por 23 itens e mede o estresse ocupacional através de três dimensões: Pressão no Trabalho, Controle Sobre as Atividades e Apoio Social. Esse instrumento permite a identificação de condições estressantes que afetam a produtividade e a saúde dos trabalhadores. O BAI contém 21 itens que avaliam a gravidade da ansiedade, abordando sintomas físicos e emocionais comuns, sendo amplamente utilizado para diagnóstico e acompanhamento terapêutico. Por fim, a Escala de *Hardiness*, composta por 45 itens, mede a resiliência psicológica através de três componentes, como Controle, Comprometimento e Desafio, avaliando a capacidade de adaptação dos indivíduos a situações estressantes. Esses instrumentos são fundamentais na pesquisa em saúde mental e na prática clínica, fornecendo dados essenciais para a elaboração de estratégias de intervenção e promoção da saúde no ambiente de trabalho.

A utilização desses instrumentos foi essencial para estabelecer sua validade convergente-discriminante. Esses instrumentos, reconhecidos e amplamente utilizados na literatura, compartilham semelhanças na avaliação de aspectos psicológicos e emocionais relacionados ao bem-estar, estresse e resiliência, permitindo verificar se o novo instrumento mede construtos similares (validade convergente) ou distintos (validade discriminante). Por exemplo, a correlação positiva entre o novo instrumento e o ProQOL-BR pode indicar que ele efetivamente mede a QVP, enquanto uma correlação negativa com a EET pode demonstrar sua capacidade de discriminar entre bem-estar e estresse ocupacional. Da mesma forma, comparações com o BAI e a Escala de *Hardiness* podem reforçar a validade do novo instrumento, ao mostrar relações consistentes com medidas de ansiedade e resiliência. Esses instrumentos estabelecem um critério robusto para avaliar a precisão e a especificidade do novo instrumento, garantindo que ele seja uma ferramenta confiável e eficaz na mensuração dos construtos psicológicos em questão.

O ProQOL-BR foi avaliado quanto à sua validade e confiabilidade, obedecendo três etapas: 1) Validação de construto por meio de análise fatorial exploratória (AFE), ratificada pela análise fatorial confirmatória (AFC) e validade convergente, estimada pela confiabilidade composta (CC). A validação de construto pretende verificar se a estrutura do instrumento consegue medir o construto teórico a que pretende; 2) Validação de critério concorrente com teste de hipóteses Pearson. A avaliação de critério procura validar se o instrumento quantifica a força e a natureza da relação entre as medidas do instrumento e os critérios externos, fornecendo evidências sobre a validade do instrumento em diferentes contextos e períodos de aplicação; 3) Análise de confiabilidade, por meio da análise de consistência interna alfa de Cronbach. A análise de confiabilidade pretende verificar se resultados são consistentes e estáveis, aumentando a confiança na interpretação das pontuações obtidas pelos participantes.

### Análise dos resultados e estatística

Para a validação do construto, utilizou-se a AFC para ratificar a estrutura dimensional extraída pela AFE. Como indicador da qualidade de ajuste local, foi utilizado o peso fatorial (λ≥0,40)^(15)^ e, para avaliar a qualidade do ajuste global do modelo de medida ajustado, foram utilizados a razão qui-quadrado e graus de liberdade (χ2/g.1), o índice de qualidade de ajuste (GFI), o índice de Tucker-Lewis (TLI), o índice de ajuste comparativo (CFI) de Bentler e a raiz do erro quadrático médio de aproximação (RMSEA). Os parâmetros considerados para avaliar o ajuste global do modelo foram λ2/g.1< 5, CFI, GFI, TLI≥0,9 e RMSEA < 0,10^(15)^, e para avaliar a validade convergente, utilizou-se CC≥0,70^(16)^.

A validade de critério concorrente propôs-se a averiguar se o ProQOL-BR era capaz de verificar maior incidência de Fadiga por Compaixão nos profissionais de enfermagem, que foram classificados com maior valor para o BAI, maior valor para a EET e com as incidências da Escala de *Hardiness*. Essa validação foi dada por meio do teste de hipóteses Pearson.

A análise de confiabilidade foi conduzida por meio da análise da consistência interna, utilizando o coeficiente alfa de Cronbach, e da correlação de item/total, a partir da matriz de correlação de todos os itens do instrumento. Considerou-se como intervalo ideal de valores de alfa entre 0,7 e 0,9^(17)^.

## RESULTADOS

Participaram deste estudo 490 profissionais de enfermagem. A análise descritiva do ProQOL-BR foi feita pela distribuição de frequências absolutas e relativas, além da classificação média das três dimensões tendo como base os 28 itens do instrumento (Tabela 1). Os profissionais apresentaram média Fadiga por Compaixão.

**Tabela 1:** Média dos escores dos fatores do *Professional Quality of Life Scale-Brazil* dos profissionais de enfermagem que atuam em serviços hospitalares de maior complexidade do norte de Minas, Montes Claros, Minas Gerais, Brasil, 2024 (N=490)

Fatores	Itens	M
Satisfação por Compaixão	Q1 - Sinto-me feliz	3,99
	Q3 - Sinto satisfação por poder cuidar das pessoas	4,42
	Q6 - Sinto-me revigorado depois de trabalhar com aqueles que cuido	3,56
	Q12 - Gosto do meu trabalho de cuidador	4,39
	Q16 - Estou satisfeito com a forma como sou capaz de manter o uso das técnicas e protocolos de cuidado	4,14
	Q17 - Sou a pessoa que sempre quis ser	3,81
	Q18 - O meu trabalho faz-me sentir satisfeito	3,96
	Q20 - Tenho pensamentos e sentimentos felizes acerca daqueles que cuido e de como poderia cuidar	4,00
	Q22 - Acredito que posso fazer a diferença através do meu trabalho	4,22
	Q24 - Orgulho-me daquilo que posso fazer para cuidar	4,36
	Q27 - Tenho pensamentos de que sou um sucesso como cuidador	3,42
	Q30 - Estou feliz por ter escolhido este trabalho	4,36
Estresse Traumático Secundário	Q5 - Assusto-me ou fico surpreendido com sons inesperados	2,55
	Q7 - Sinto que é difícil separar minha vida pessoal da minha vida como cuidado	2,28
	Q8 - Perco o sono revivendo experiências traumáticas de uma pessoa que cuido	1,50
	Q9 - Penso que posso ter sido afetado pelo estresse traumático daqueles que eu cuido	1,81
	Q10 - Sinto-me acurralado pelo meu trabalho como cuidador	1,63
	Q11 - Por causa da minha ajuda, já me senti no limite em relação à várias coisas	2,25
	Q13 - Sinto-me deprimido por causa das experiências traumáticas das pessoas que eu cuido	2,03
	Q14 - Sinto-me como se estivesse a viver o trauma de alguém que tenha cuidado	1,81
	Q23 - Evito certas atividades ou situações porque me fazem relembrar experiências assustadoras vividas pelas pessoas que cuido	1,72
	Q25 - Como resultado do meu cuidado, tenho pensamentos invasivos e assustadores	1,48
*Burnout*	Q19 - Sinto-me exausto por causa do meu trabalho como cuidador	2,54
	Q21 - Sinto-me esgotado porque minha carga de trabalho parece nunca acabar	2,48
	Q26 - Sinto-me limitado pelo trabalho	2,53

A dimensão Satisfação por Compaixão obteve uma pontuação média geral de 59,48 pontos, classificando-se em “média Satisfação por Compaixão”. No grupo com fatores mais baixos, destacou-se “Q27 - Tenho pensamentos de que sou um sucesso como cuidador” (M= 3,42). As questões que mais apresentaram fatores elevados foram “Q3 - Sinto satisfação por poder cuidar das pessoas” (M= 4,42), seguidas de “Q12 - Gosto do meu trabalho de cuidador” (M= 4,39), “Q24 - Orgulho-me daquilo que posso fazer para cuidar” e “Q30 - Estou feliz por ter escolhido este trabalho” (ambas com M=4,36).

A dimensão Estresse Traumático Secundário obteve uma pontuação média geral de 19,06 pontos, classificando-se em “baixo Estresse Traumático Secundário”. As questões que mais apresentaram fatores elevados foram “Q5 - Assusto-me ou fico surpreendido com sons inesperados” (M= 2,55), “Q7 - Sinto que é difícil separar minha vida pessoal da minha vida como cuidador” (M=2,28) e “Q11 - Por causa da minha ajuda, já me senti no limite em relação à várias coisas” (M= 2,25). No grupo com fatores mais baixos, destacam-se “Q25 - Como resultado do meu cuidado, tenho pensamentos invasivos e assustadores” (M= 1,48) e “Q8 - Perco o sono revivendo experiências traumáticas de uma pessoa que cuido” (M= 1,50).

A dimensão *Burnout* obteve uma pontuação média geral de 7,55 pontos, classificando-se em “médio *Burnout*”. A questão que mais apresentou fator elevado foi “Q19 - Sinto-me exausto por causa do meu trabalho como cuidador” (M= 2,54). A questão com fator mais baixo foi “Q21 - Sinto-me esgotado porque minha carga de trabalho parece nunca acabar” (M= 2,48). Ressalta-se que a variação nesta dimensão foi muito pouca (0,06 pontos), representando, assim, um equilíbrio entre os fatores para *Burnout*.

O modelo proposto ratificado pela AFC apresentou qualidade adequada de ajuste local pelos pesos fatoriais (λ≥ 0,40) em 25 questões (Figura 1). Além disso, três questões - Q2 (λ=0,09), Q4 (λ=0,35) e Q15 (λ=0,12) - do ProQOL-BR foram retiradas, pois apresentaram baixas cargas fatoriais para profissionais de enfermagem. O modelo também apresentou qualidade de ajuste global aceitável pela razão qui-quadrado e graus de liberdade (χ2/g.1=2,51) e adequado para os parâmetros de CFI (0,923), GFI (0,902), TLI (0,914) e RMSEA (0,042). Na avaliação da validade convergente, o modelo proposto também se mostrou adequado, com CC=0,89.

**Figura 1:** Modelo do *Professional Quality of Life Scale-Brazil* ratificado pela análise fatorial confirmatória, Montes Claros, Minas Gerais, Brasil, 2024 (N=490)

**--------**Os resultados da confiabilidade do ProQOL-BR revelam que a consistência interna obtida de alfa de Cronbach padronizado foi de 0,761 para toda a amostra e de 0,768 a 0,860 para as dimensões do instrumento. Os valores de alfa de Cronbach, caso determinado item fosse excluído, mostraram-se adequados em 26 itens, já que todos os itens apresentaram valores de 0,801 a 0,868. Dois itens da dimensão *Burnout* apresentaram índices próximos do adequado caso fossem excluídos (Q-19 0,631 e Q-21 0,629), mas foram mantidos devido ao índice e ao número de itens da dimensão. As correlações do item total corrigido realizadas por meio do coeficiente de correlação de Pearson foram fracas para o item “Q2 - Estou preocupado com mais de uma pessoa que eu cuido” (r=0,127) e fortes para o item “Q10 - Sinto-me acurralado pelo meu trabalho como cuidador” (r=0,667) (Tabela 2).

**Tabela 2:** Análise da consistência interna do *Professional Quality of Life Scale-Brazil* por meio do coeficiente alfa de Cronbach e correlação item total corrigido, Montes Claros, Minas Gerais, Brasil, 2024 (N=490)

Dimensões do ProQOL-BR	Item do ProQOL-BR	Alfa de Cronbach (IC 95%)	Alfa de Cronbach se o item for retirado	Coreelação Item-Total
Satisfação por Compaixão	Q1 - Sinto-me feliz	0,845	0,831	0,571
	Q3 - Sinto satisfação por poder cuidar das pessoas		0,830	0,638
	Q6 - Sinto-me revigorado depois de trabalhar com aqueles que cuido		0,831	0,546
	Q12 - Gosto do meu trabalho de cuidador		0,827	0,661
	Q16 - Estou satisfeito com a forma como sou capaz de manter o uso das técnicas e protocolos de cuidado		0,833	0,528
	Q17 - Sou a pessoa que sempre quis ser		0,831	0,539
	Q18 - O meu trabalho faz-me sentir satisfeito		0,824	0,664
	Q20 - Tenho pensamentos e sentimentos felizes acerca daqueles que cuido e de como poderia cuidar		0,826	0,660
	Q22 - Acredito que posso fazer a diferença através do meu trabalho		0,830	0,592
	Q24 - Orgulho-me daquilo que posso fazer para cuidar		0,830	0,611
	Q27 - Tenho pensamentos de que sou um sucesso como cuidador		0,840	0,410
	Q30 - Estou feliz por ter escolhido este trabalho		0,826	0,638
Estresse Traumático Secundário	Q5 - Assusto-me ou fico surpreendido com sons inesperados	0,860	0,860	0,427
	Q7 - Sinto que é difícil separar minha vida pessoal da minha vida como cuidado		0,863	0,429
	Q8 - Perco o sono revivendo experiências traumáticas de uma pessoa que cuido		0,847	0,582
	Q9 - Penso que posso ter sido afetado pelo estresse traumático daqueles que eu cuido		0,830	0,758
	Q10 - Sinto-me acurralado pelo meu trabalho como cuidador		0,838	0,667
	Q11 - Por causa da minha ajuda, já me senti no limite em relação à várias coisas		0,847	0,565
	Q13 - Sinto-me deprimido por causa das experiências traumáticas das pessoas que eu cuido		0,834	0,709
	Q14 - Sinto-me como se estivesse a viver o trauma de alguém que tenha cuidado		0,839	0,653
	Q23 - Evito certas atividades ou situações porque me fazem relembrar experiências assustadoras vividas pelas pessoas que cuido		0,855	0,463
	Q25 - Como resultado do meu cuidado, tenho pensamentos invasivos e assustadores		0,849	0,542
*Burnout*	Q19 - Sinto-me exausto por causa do meu trabalho como cuidador	0,768	0,631	0,657
	Q21 - Sinto-me esgotado porque minha carga de trabalho parece nunca acabar		0,629	0,654
	Q26 - Sinto-me limitado pelo trabalho		0,801	0,506
Escore total		0,761		

**----------**A validade de critério concorrente do ProQOL-BR mostrou-se favorável para profissionais de enfermagem (Tabela 3). A significância das correlações foi significativa (0,001), com algumas magnitudes média e a maioria forte. Todas as dimensões do ProQOL-BR com as dimensões da Escala de *Hardiness* (Compromisso e Controle) apresentaram magnitude forte de correlação, variando de 0,335 a 0,484. A correlação das dimensões do ProQOL-BR com a dimensão da Escala de *Hardiness* (Desafio) mostrou-se de magnitude média, variando de 0,202 a 0,268. A correlação das dimensões do ProQOL-BR com o EET mostrou-se com magnitude forte de correlação, variando de 0,350 a 0,597. A correlação das dimensões do ProQOL-BR com o BAI mostrou-se com magnitude forte de correlação nas dimensões Estresse Traumático Secundário e *Burnout*, variando de 0,426 a 0,493, com magnitude média de correlação na dimensão Satisfação por Compaixão, com valor de 0,218.

**Tabela 3:** Correlações de Pearson entre as medidas, obtidas pelos instrumentos *Professional Quality of Life Scale-Brazil*, Escala de *Hardiness*, Escala de Estresse no Trabalho e Inventário de Ansiedade de Beck

Escalas	ProQOL-BR		
	D1 (Satisfação) *r (p-valor)*	D2 (Estresse) *r (p-valor)*	D3 (*Burnout*) *r (p-valor)*
Escala de *Hardiness* (Compromisso)	0,484 (<0,001)	-0,462 (<0,001)	-0,469 (<0,001)
Escala de *Hardiness* (Controle)	0,405 (<0,001)	-0,394 (<0,001)	-0,335 (<0,001)
Escala de *Hardiness* (Desafio)	0,210 (<0,001)	-0,202 (<0,001)	-0,268 (<0,001)
Escala de Estresse no Trabalho total	-0,350 (<0,001)	0,470 (<0,001)	0,597 (<0,001)
Inventário de Ansiedade de Beck	-0,218 (<0,001)	0,493 (<0,001)	0,426 (<0,001)

--------------

## DISCUSSÕES

O presente estudo possibilitou resultados que demonstraram as validades de construto, confiabilidade e critério do ProQOL-BR na população de profissionais de enfermagem atuantes em unidades de assistência hospitalar de alta complexidade. Esses resultados apresentaram-se consistentes e confiáveis. O instrumento é importante para direcionar a detecção de AE neste público, além de servir para o alcance da meta ODS 8 (Trabalho descente e crescimento econômico), visando proteção dos direitos trabalhistas e promoção de ambientes de trabalho mais seguros para os profissionais de enfermagem^(2)^. A literatura nacional aponta que a enfermagem é protagonista de qualquer empresa de saúde. Suas entregas impactam custos, satisfação dos clientes e, principalmente, o objetivo final desta empresa e o reestabelecimento das condições de saúde de quem procura o serviço^(18)^.

Este estudo apontou um alto índice de participação na pesquisa. Além disso, 82% dos profissionais convidados responderam aos questionários, demonstrando que a temática é de interesse e relevância para esta categoria. Destaca-se que, principalmente após o período pandêmico da COVID-19, os profissionais de enfermagem voltaram-se muito aos olhares de preocupações com a sua situação emocional^(19)^. A preocupação com as questões de AE torna-se fundamental para a criação de um sistema de saúde eficaz e humano.

O ProQOL-Br possibilita várias análises importantes acerca da qualidade de vida no trabalho dos profissionais de enfermagem, resultando em uma análise global de Fadiga por Compaixão. Na dimensão Satisfação por Compaixão, os resultados apontaram “média Satisfação por Compaixão”. Houve um equilíbrio resultante de índices baixos em relação ao sucesso da consequência dos seus cuidados e índices altos em relação à satisfação de ser profissional de enfermagem. Esse resultado pode estar relacionado à gravidade dos pacientes a que esses profissionais prestam assistência, um ambiente carregado de óbitos e cuidados paliativos, mas também que oportuniza um “orgulho” em poder utilizar seus conhecimentos e habilidades para fazer o melhor para o ser humano em situações tão críticas de saúde^(20)^.

No tocante ao Estresse Traumático Secundário, que se refere a pensamentos invasivos e assustadores resultantes do cuidado prestado, o instrumento apontou “baixo estresse secundário”, um dos questionamentos que deram resultados mais baixos nos profissionais de enfermagem do estudo. O Estresse Traumático Secundário foi desenvolvido para mensurar sintomas especificamente em profissionais que fornecem cuidados. Resultados semelhantes a este estudo de Estresse Traumático Secundário foram indicados em pesquisa de validação que apontou resultados com bombeiros e paramédicos^(21)^.

A dimensão *Burnout* apresentou resultados de “médio *burnout*”. Estudo^(22)^ utilizou o instrumento *Maslach Burnout Inventory* (MBI), validado no Brasil, para avaliar essa condição em profissionais de enfermagem que atuam em terapia intensiva. Esse estudo também apontou uma baixa média para o *burnout* em sua população. O mesmo estudo relembra que os índices de *burnout* em profissionais de enfermagem brasileiros são menores em comparação aos europeus.

Os profissionais de enfermagem deste estudo não apresentaram Fadiga por Compaixão. Os resultados se assemelham aos encontrados em estudo no estado do Paraná^(23)^. Esses dados podem estar relacionados à resiliência no trabalho. Nos resultados e discussões deste estudo no Paraná, a resiliência demonstrou ser um fator de proteção às variáveis de saúde mental, como a presença de distúrbios psíquicos menores, desgaste emocional e despersonalização^(23)^.

A AFC apontou ajuste favorável adequado do instrumento em relação à população de profissionais de enfermagem que atuam em terapia intensiva. As cargas fatoriais com melhores ajustes apontaram que os profissionais se sentem felizes em ter escolhido a enfermagem como profissão, mas ao mesmo tempo reconhecem o quanto são afetados pelo estresse do processo de cuidar, o que causa exaustão. Os resultados são os mesmos de estudo das AE de profissionais de enfermagem que atuaram em terapias intensivas em pacientes com COVID-19^(24)^. Esses profissionais percebem-se exaustos emocionalmente, bem como seus colegas de equipe. A origem da exaustão emocional relaciona-se à instabilidade clínica, elevado número de óbitos e frequência das mudanças institucionais.

Os resultados mostraram a confiabilidade do instrumento para todas as suas dimensões. Os valores de maior destaque apontaram que os profissionais de enfermagem atuantes em terapia intensiva têm dificuldade em separar a vida pessoal da profissional, sendo que, em ambientes fora do trabalho, evitam certas atividades ou situações que possam relembrar experiências desagradáveis que tiveram com os seus pacientes.

As análises apontaram validade de critério concorrente, sendo o ProQOL-BR capaz de verificar maior incidência de Fadiga por Compaixão nos profissionais de enfermagem que foram classificados com maior valor para ansiedade (BAI) e maior valor para estresse no trabalho (EET). A ansiedade e o estresse foram realçados neste estudo, assim como aponta a literatura. Em estudo brasileiro^(25)^, a grande maioria (75%) dos profissionais apresentou sofrimento mental, destacando sintomas de depressão, ansiedade e estresse na execução das suas rotinas de trabalho.

A validade de critério também se mostrou favorável com o instrumento sobre personalidade *hardiness*. Os dados obtidos com esse instrumento mostraram que, quanto maior o comportamento de enfrentamento perante situações de adversidade, melhores os índices relacionados à Satisfação por Compaixão e piores os índices para Estresse e *Burnout*. Os profissionais deste estudo apresentaram índices satisfatórios para personalidade *hardiness* em suas três dimensões, o que também foi encontrado em estudo^(26)^ realizado com profissionais de uma empresa de alta complexidade, demonstrando que a personalidade *hardiness* influencia direta e indiretamente a saúde e o bem-estar, promovendo o uso de recursos sociais e alterando o estresse autopercebido, reduzindo, assim, a tensão no trabalho em saúde.

### Limitações do estudo

Apesar da relevância do instrumento elaborado, é preciso considerar sua limitação. Poucos estudos nacionais aplicaram a metodologia proposta na validação do ProQOL-Br^(8)^ para interpretação dos seus resultados. A partir desses resultados, destaca-se a necessidade de atualização da análise de valor pelos profissionais de enfermagem.

### Contribuições para as áreas da enfermagem, saúde ou políticas públicas

Espera-se que o uso desse instrumento possibilite a implantação de ações voltadas para processos de intervenção de AE prejudiciais em profissionais de enfermagem, como também a identificação das potencialidades e fragilidades dos processos. Além disso, espera-se que a aplicação dessa ferramenta auxilie gestores e profissionais de saúde na estruturação de suas políticas de recursos humanos e em todas as relações interpessoais existentes em um ambiente tão complexo e recheado de nuances distintas como o cuidado de enfermagem a pacientes críticos.

## CONCLUSÕES

O desenvolvimento metodológico para análise e validação do ProQOL-Br para os profissionais de enfermagem atuantes em empresas assistenciais de alta complexidade tornou possível o entendimento de um tema de difícil mensuração: as questões emocionais. Esse entendimento e validação contribui significativamente para qualquer intervenção na temática.

O instrumento encontra-se validado no que concerne ao seu conteúdo, critério e confiabilidade. A partir do instrumento original, ProQOL-BR, foram retiradas três questões, ficando o instrumento final com 25 questões. Ressalta-se que novas análises devem ser avaliadas. O instrumento encontra-se disponível para utilização em estudos, e análises posteriores poderão ser úteis no seu aprimoramento.

Para a avaliação de AE em profissionais de enfermagem brasileiros, recomenda-se a utilização desse instrumento, uma vez que sua validação ocorreu com profissionais em momento assistencial pandêmico, e o número questões torna a aplicação rápida e objetiva.
